# Variability measurements provide additional value to shear wave elastography in the diagnosis of pancreatic cancer

**DOI:** 10.1038/s41598-021-86979-5

**Published:** 2021-04-01

**Authors:** Masakatsu Yoshikawa, Takuya Ishikawa, Eizaburo Ohno, Tadashi Iida, Kazuhiro Furukawa, Masanao Nakamura, Takashi Honda, Masatoshi Ishigami, Fumie Kinoshita, Hiroki Kawashima, Mitsuhiro Fujishiro

**Affiliations:** 1 Department of Gastroenterology and Hepatology, Nagoya University Graduate School of Medicine, 65 Tsuruma-cho, Showa-ku, Nagoya, Aichi 466-8550 Japan; 2 Data Coordinating Center, Department of Advanced Medicine, Nagoya University Hospital, 65 Tsuruma-cho, Showa-ku, Nagoya, Aichi 466-8550 Japan; 3 Department of Endoscopy, Nagoya University Hospital, 65 Tsuruma-cho, Showa-ku, Nagoya, Aichi 466-8550 Japan

**Keywords:** Gastroenterology, Medical research

## Abstract

Shear wave elastography (SWE) is a technique to non-invasively and quantitatively evaluate tissue stiffness. We aimed to investigate whether we can differentiate pancreatic cancer (PC) from normal pancreatic parenchyma (NPP) by SWE using transabdominal ultrasound. We investigated a total of 106 patients (84 with NPP and 22 with PC) whose pancreatic elastic modulus was measured by two-dimensional SWE (2D-SWE). Intra-rater reliability in this study was examined, and three measurements were sufficiently reliable. There were no differences between the two groups in factors that could affect SWE measurements. The median value of the elastic modulus was 5.70 kPa in the PC patients and 5.66 kPa in the NPP group, which was not significantly different (*P* = 0.785). On the contrary, the range was 8.64 kPa and 4.72 kPa, with a significantly greater range in the PC patients (*P* = 0.001). In conclusion, the median elastic modulus measured by 2D-SWE was not significantly different between PC and NPP, and evaluating the obtained elastic modulus itself is not useful in differentiation. However, the variability was significantly greater in PC than in NPP. Evaluating the range of elasticities will provide additional information in SWE, which may be useful in the diagnosis of PC.

## Introduction

Ultrasonic elastography is a technique for measuring the stiffness of tissues. Shear wave elastography (SWE) is a recently developed technique to measure the shear wave speed (SWS) within a region of interest (ROI) generated by push pulses by repeatedly emitting exploratory ultrasound pulses to objectively and quantitatively evaluate tissue stiffness^1–4^. This enables a non-invasive and simple evaluation of tissue stiffness. SWE has been reported to be useful in the evaluation of liver fibrosis and steatosis, and its clinical significance is gradually becoming clear^5,6^. Recently, SWE has also been reported to be useful in the pancreas, such as for the diagnosis of fibrosis in chronic pancreatitis^7^. However, few SWE-related reports for the diagnosis of pancreatic cancer (PC) are available. Although PC often forms a stiff mass^8^, whether the tissue stiffness of PC can be correctly evaluated by SWE is not clear. The aim of this study was to determine whether we could differentiate PC from normal pancreatic parenchyma (NPP) by SWE measured using a state-of-the-art ultrasound instrument, the EPIQ 7G (Philips Medical Systems, Bothell, WA).

## Materials and methods

### Study design

This was a single-center, case–control study. This research was conducted in strict compliance with the Declaration of Helsinki and in accordance with the ethical guidelines for medical research involving human subjects. Written informed consent was obtained from all patients in this study. The study was conducted with the approval of the Ethics Committee of Nagoya University Hospital (approval number: 2014-0399).

### Patients

We retrospectively reviewed 98 NPP, 22 PC and 4 mass-forming pancreatitis (MFP, 2 chronic pancreatitis and 2 type 1 autoimmune pancreatitis) patients who underwent SWE using the EPIQ 7G at Nagoya University Hospital between January 2019 and October 2019. In all PC patients, a mass was detected in the pancreas on CT within 2 weeks before transabdominal ultrasound (US). Using the CT image as a guide, all of the lesions could be identified on US, and SWE measurement was possible in all 22 cases. A pathological diagnosis was obtained in all PC cases by surgery (n = 13) or endoscopic ultrasound-guided fine-needle aspiration (EUS-FNA) (n = 9), and the histological type was adenocarcinoma in all patients. The median tumour diameter of PC was 31 mm (20.8–37.0), and the locations of the tumours were the head in 13 cases, the body in 7 cases, and the tail in 2 cases, with distant metastasis identified in 5 cases (Table 1).

**Table 1:** The site, size, metastasis site and pathological diagnostic modality of pancreatic cancer.

	No	%
**Site of the cancer**		
Head, n	13	59.1
Body, n	7	31.8
Tail, n	2	9.1
Size of the cancer, median (IQR), mm	31 (20.8–37.0)	
**Distant metastasis**		
Liver, n	0	0
Lung, n	3	13.6
Lymph nodes, n	2	9.1
**Pathological diagnosis**		
Operation, n	13	59.1
EUS-FNA, n	9	40.9

*IQR* interquartile range, *EUS-FNA* endoscopic ultrasound-guided fine needle aspiration.

The 98 NPP patients were enrolled from among the 332 patients who underwent US using the EPIQ 7G during the study period and had no history of pancreatic disease and no abnormalities on CT or endoscopic ultrasound (EUS) imaging within 3 months before US, such as cyst or expansion or narrowing of the pancreatic duct. In these 98 patients, the pancreatic parenchyma of the body could be clearly depicted on US in 84 cases, and SWE measurement was possible in all of these cases.

The 4 MFP patients were also enrolled from among the 332 patients shown above. All 4 patients had CT and EUS-FNA at the initial assessment, and the final diagnosis was made with 1 follow-up of at least 12 months. The locations of the lesions were the head in 3 cases and the body in 1 case.

Finally, SWE measurement was possible in a total of 110 patients (22 PC, 84 NPP and 4 MFP cases), and the SWE values were evaluated in these cases. The examination was performed by one gastroenterologist, who has experience with more than 3000 transabdominal ultrasounds and is a specialist in the Japanese Society of Ultrasound Medicine, and one gastroenterologist who is a full member of the Japanese Society of Ultrasound Medicine.

### SWE measurement

**Figure 1:**
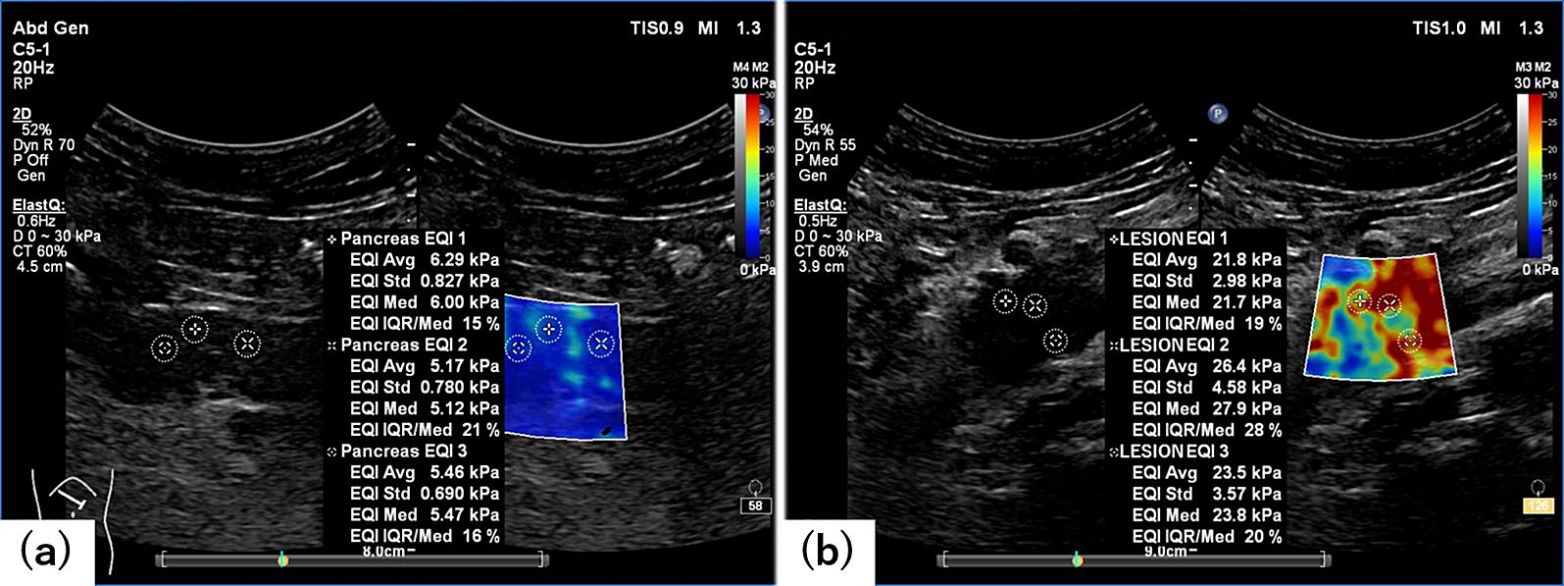
Shear wave elastography (SWE) images and measurements. (**a**) SWE images of normal pancreatic parenchyma. The left side image shows a B-mode image, and the right side image shows a stiffness map. Multiple regions of interest can be set anywhere in the stiffness map, and the SWE results are described in the center of the display. (**b**) SWE images of pancreatic cancer.

The EPIQ 7G instrument was used in all cases. The transducer was a convex transducer, C5-1. The SWE was measured in the ElastQ mode onboard the instrument. The ElastQ mode is the 2-dimensional SWE (2D-SWE) on the EPIQ7G, which allows the size of the area of interest to be extended to a maximum of 6 cm × 5 cm, and the measurement area can be set to any area of the colour-mapped stiffness map. When performing SWE with ElastQ mode, a confidence map is also generated within the area of interest using smart analysis of echo and shear wave signals. The confidence map reflects a value for each pixel, providing an indication of quality across the stiffness map. The areas with low confidence values are shown as colour defects in the stiffness map. Several factors can lower the confidence value: tissue areas with blood flow (vessels), low echogenicity (such as the gallbladder), low shear wave strength (as when scanning deep in a technically difficult patient), or with large tissue motion (as with no breath pause). In this study, interquartile range (IQR)/median, which is a quantitative measurement, rather than the confidence map, which is a qualitative measurement, was used as an indicator of reliability. The SWE is saved as a video (raw data) lasting a few seconds and can be rewound to measure the SWE from any still image. In this study, a 6-s video was saved uniformly. The patient was instructed to hold his or her breath when the evaluation site was clearly depicted in B-mode and was within the area of interest, and the video was saved. In the NPP group, the area of interest was set in the body of the pancreas, which could be clearly described, and in the PC and the MFP group, the area of interest was set where the maximum diameter of the lesion could be clearly described (Fig. 1). Three videos were stored at the same site, and SWE was measured after the examination. For the measurement, one still image was manually selected from each movie with the least colour defect of the target area in the stiffness map. The measurement was performed with a circular ROI, and the diameter was unified to 5 mm. Schellhaas et al. have evaluated the influenced of ROI size in the assessment of liver stiffness using 2D-SWE, and concluded that the size of the ROI (5, 10, 20 mm) seemed to be of minor importance with comparable results and it was more important to perform multiple measurements^9^. Since the pancreas is smaller than the liver, we set the ROI size to 5 mm so that we can measure at multiple locations. As there is no clear consensus on the setting of SWE measurements in the pancreatic region, we set the threshold to 60%, which is the standard setting of liver SWE measurements. The ROI was set at three locations per image where the IQR/median was 30% or less, and if multiple locations (10 locations) did not result in 30% or less, the three locations with the lowest IQR/median were selected. The SWE measurement screen also displays a colour map, but location of the ROI was set using the IQR/median as an indicator, regardless of the colour distribution. A total of nine measurements were stored for each of the three stored videos in the same way, and the median of the nine measurements (kPa) was defined as the elastic modulus of the evaluation site.

### Examination items and statistical analysis

The reproducibility and reliability of ultrasonic elastography is a challenging issue, and it has been reported that the measured values differ depending on the instrument^10^. It has also been reported that values vary not only by disease but also by background factors such as age, BMI, and distance from the probe to the measurement target^11^. Thus, this study was performed in the following five steps.

1. Normal values of SWE and intraclass correlation coefficient (ICC) of SWE measurement in the pancreas in ElastQ mode
  The ICC was calculated for the NPP group, and the intra-rater reliability was evaluated. In this study, three still images were selected from the video saved in ElastQ mode, three SWE measurements were taken at three locations per image, and a total of nine measurements were evaluated. In the intra-rater reliability study, the median value of three measurements in the same image was used as the value per image, and ICC (1,1) and ICC (1,3) were calculated to verify whether the SWE could be reliably measured by three images. The elastic modulus calculated by this method was defined as the normal value of the pancreas.
2. Differences in clinical background between the NPP group and PC group
  Age, sex, body mass index (BMI), medical history (hypertension, dyslipidaemia, diabetes), the distance between the pancreatic parenchyma and the body surface, the distance between the PC tumour area and the body surface, and blood biochemical tests were compared between NPP and PC patients. The distance between the pancreatic parenchyma and the body surface was set as the distance from the proximal edge of the pancreatic body, which is the thickest part of the body where SWE is measured, to the probe. Similarly, the distance between PC tumours and the body surface was defined as the distance from the proximal edge of the tumour to the probe at the site of SWE measurement.
3. Comparison of elastic modulus between the NPP group and PC group
  Based on the SWE values in the normal pancreas obtained in (1) above, the median SWE values of the NPP group and PC group were compared.
4. Comparison of the variability of elastic modulus between the NPP group and PC group
  Because of the expected high tissue heterogeneity in PC^12^, we calculated the minimum values, maximum values and ranges (the difference between the maximum and minimum values) within the measurements as well as the median values obtained from multiple measurements in each patient and compared the variability between the NPP group and PC group.
5. Comparison of elastic modulus and its variability between the PC group and MFP group
  The median SWE values and the variability of the PC group and MFP group ware compared.

Fisher's exact test was used for categorical variables, and the Mann–Whitney U test or the Kruskal–Wallis test was used for continuous variables; the ICCs (1,1) and (1,k) were calculated using the classification of Shrout and Fleiss^13^. A *P* value of less than 0.05 was considered statistically significant. We analysed the receiver operating characteristic curves for the mean and range values of the elastic modulus. SPSS ver. 26 (SPSS, Inc., Tokyo, Japan) was used for all analyses.

## Results

**Figure 2:**
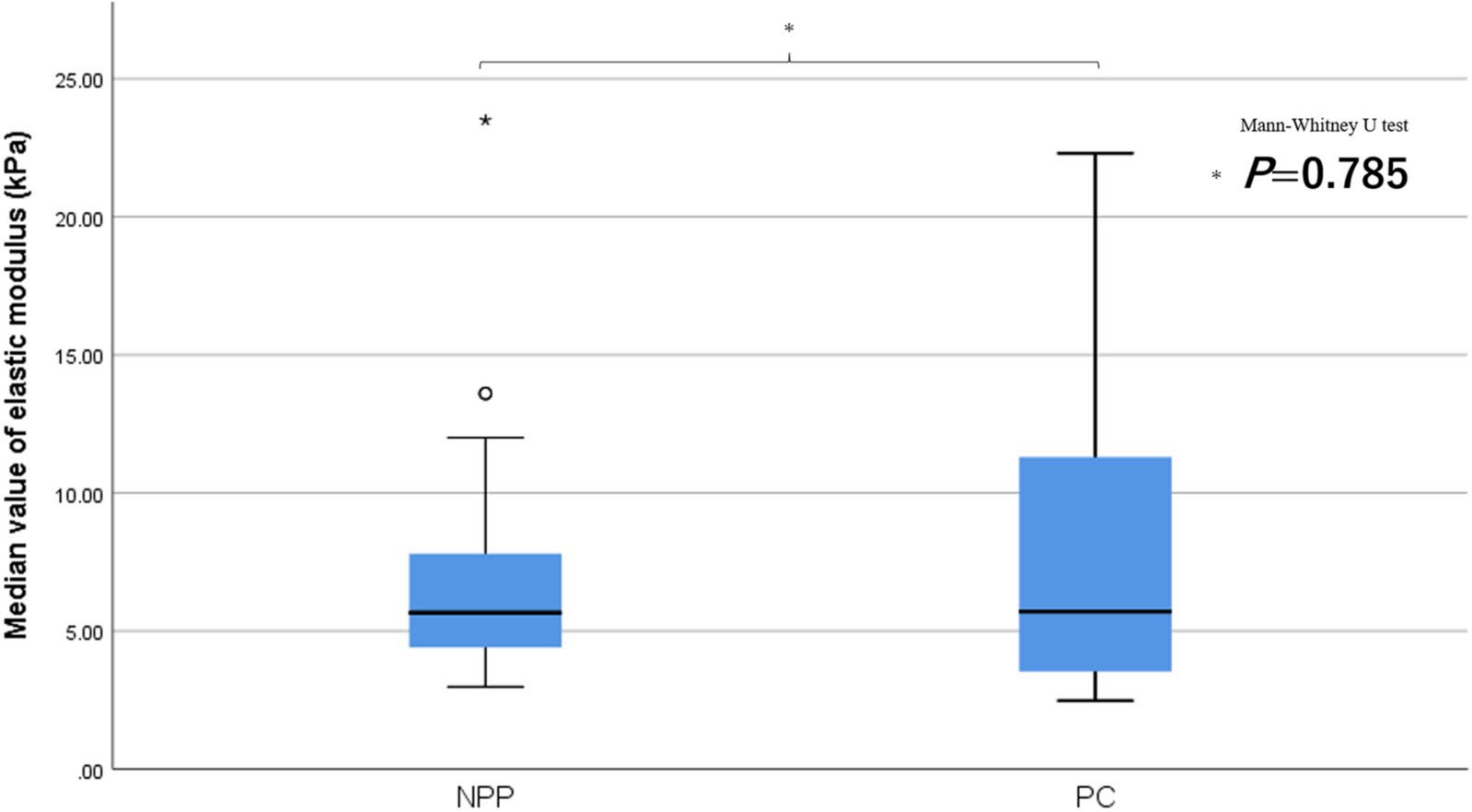
Comparison of elastic modulus between normal pancreatic parenchyma (NPP) group and pancreatic cancer (PC) group. The median values of each group are compared using a t-test, and the error bars show interquartile range.

**Figure 3:**
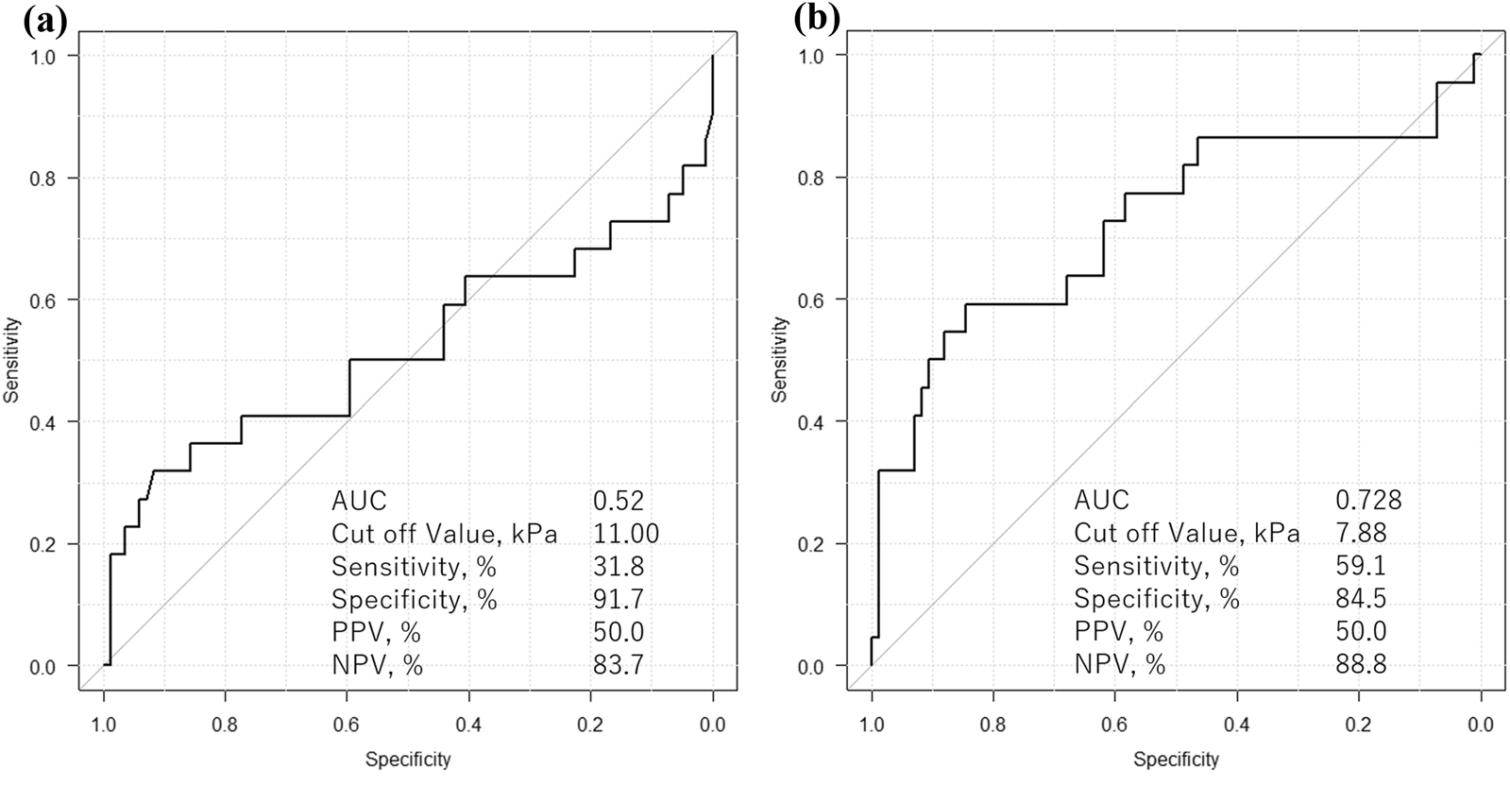
The receiver operating characteristic curve for the diagnostic ability of the mean (**a**) and range (**b**) values for pancreatic cancer. (**a**) With a cut-off value of 11.00 kPa, the sensitivity, specificity, positive predictive value (PPV), and negative predictive value (NPV) of SWE for the diagnosis of pancreatic cancer were 31.8%, 91.7%, 50.0%, and 83.7%, respectively. (**b**) With a cut-off value of 7.88 kPa, the sensitivity, specificity, PPV, and NPV of SWE for the diagnosis of pancreatic cancer were 59.1%, 84.5%, 50.0%, and 88.8%, respectively.

1. In one image measurement (three measurements) in the NPP group, the ICC (1,1) was ρ = 0.668 (IQR 0.566–0.757), and the ICC (1,3) was ρ = 0.858 (0.796–0.904) (Table 2). Pancreatic elastic modulus [median (IQR)] in the NPP group determined from a total of nine measurements in three images was 5.66 (4.39–7.84) kPa. This value was compared with the elastic modulus in the PC group determined in (3).
2. Significant differences in albumin, AST, ALT, ALP, γGTP, CA19-9, and a history of diabetes were found between the NPP group and the PC group, but no differences were observed between the two groups in factors that could affect SWE measurement, such as age, BMI, and the distance between the probe and the measurement target (Table 3).
3. The median elastic modulus of the PC group was 5.70 (3.48–11.55) kPa, which was not significantly greater than that of the NPP group (*P* = 0.785) (Fig. 2). The ROC curve using the median value of nine measurements gave an AUC = 0.52, with a sensitivity of 31.8%, specificity of 91.7%, positive predictive value of 50.0%, and negative predictive value of 83.7% when 11.00 kPa was the cut-off value (Fig. 3a).
4. Each results is shown in Table 4. The median of the minimum values, maximum values, and range values were evaluated for one image (three measurements), two images (six measurements), and three images (nine measurements). The minimum values with 3, 6, and 9 measurements were 4.25/3.85/3.75 kPa in the NPP group and 3.92/3.21/3.05 kPa in the PC group, respectively, with significantly smaller values in the PC group with nine measurements (P = 0.040). The maximum values with three, six, and nine measurements were 6.37/7.55/8.69 kPa in the NPP group and 8.34/12.05/13.15 kPa in the PC group, respectively, with significantly larger values in the PC group with six and nine measurements (*P* = 0.009, *P* = 0.008, respectively). The range values with three, six, and nine measurements were 1.80/3.55/4.72 kPa in the NPP group and 3.40/6.83/8.64 kPa in the PC group, respectively, with significantly higher values in the PC group (*P* = 0.043, *P* = 0.003, *P* = 0.001, respectively). The ROC curve using the range value of nine measurements gave an AUC 0.728, with a sensitivity of 59.1%, specificity of 84.5%, positive predictive value of 50%, and negative predictive value of 88.8% when 7.88 kPa was the cut-off value (Fig. 3b).
5. The median elastic modulus of the MFP group was 3.94 (3.18–9.26) kPa, which was not significantly different from that of the PC group (*P* = 0.352). The range value with nine measurements was 3.41 kPa in the MFP group, which was significantly smaller than that of the PC group (*P* = 0.021). There was no significant difference in the elastic modulus between NPP and MFP (*P* = 0.177).

**Table 2:** Intra-rater reliability.

	ρ	95% CI
ICC (1,1)	0.668	0.566–0.757
ICC (1,3)	0.858	0.796–0.904

*ICC* intraclass correlation coefficients, *CI* confidence interval.

**Table 3:** Characteristics of the study population.

	NPP (n = 84)	PC (n = 22)	*P* value
Age, median (IQR)	64 (53–72.8)	70 (63.8–76)	0.064*
Sex, n (male/female)	53/31	17/5	0.460**
Weight, median (IQR), kg	58.1 (47.8–67.2)	54.2 (51.7–58.5)	0.521*
BMI, median (IQR), kg/m^2^	21.8 (19.5–24.8)	20.7 (19.2–22.7)	0.403*
**Biochemical profile**			
WBC, median (IQR), 10^3^/mm^3^	5.5 (4.2–6.7)	5.3 (4.5–6.1)	0.675*
Hb, median (IQR), mg/dL	13.9 (12.4–15.0)	13.7 (12.6–14.7)	0.392*
Plt, median (IQR), 10^3^/mm^3^	202 (168–237)	216 (158–235)	0.764*
Alb, median (IQR), g/dL	4.2 (4.0–4.4)	4.0 (3.7–4.3)	0.042*
AST, median (IQR), U/L	20 (17–25)	24 (21–52)	0.012*
ALT, median (IQR), U/L	17 (11–23)	30 (18–56)	0.002*
ALP, median (IQR), U/L	208 (170–247)	230 (199–544)	0.009*
γGTP, median (IQR), U/L	22 (16–35)	32 (17–149)	0.043*
T-Bil, median (IQR), mg/dL	0.8 (0.6–1.1)	0.9 (0.5–1.1)	0.874*
AMY, median (IQR), U/L	72 (57–99)	65 (56–82)	0.166*
CRP, median (IQR), mg/L	0.05 (0.03–0.14)	0.06 (0.04–0.17)	0.644*
Lipase, median (IQR), IU/L	33 (24–39)	34 (26–47)	0.433*
CEA, median (IQR), ng/mL	2.1 (1.2–3.3)	2.9 (1.9–3.7)	0.059*
CA19-9, median (IQR), U/mL	14.0 (7.8–25.3)	136.5 (26.5–587.5)	< 0.001*
**Past history**			
Hypertension, n (%)	14 (16.1)	6 (27.3)	0.231**
Dyslipidaemia, n (%)	17 (19.5)	7 (31.8)	0.252**
Diabetes, n (%)	12 (13.8)	9 (40.9)	0.012**
Distance from pancreatic parenchyma or tumor to probe, median (IQR), cm	3.34 (2.43–4.33)	2.95 (2.18–3.59)	0.183*

*NPP* normal pancreatic parenchyma, *PC* pancreatic cancer, *IQR* interquartile range, *BMI* body mass index, *WBC* white blood cell, *Hb* hemoglobin, *Plt* platelet, *Alb* albumin, *AST* aspartate transaminase, *ALT* alanine transaminase, *ALP* alkaline phosphatase, *γGTP* gamma-glutamyl transpeptidase, *T-Bil* total bilirubin, *AMY* amylase, *CRP* C-reactive protein, *CEA* carcinoembryonic antigen, *CA19-9* carbohydrate antigen 19-9.

*Mann–Whitney U test, **Fisher’s exact test.

**Table 4:** Comparison of elastic modulus variability between normal pancreatic parenchyma group and pancreatic cancer group.

	NPP (n = 84)	PC (n = 22)	*P* value
Minimum value per 3 areas, median (IQR), kPa	4.25 (3.38–5.71)	3.92 (3.05–11.55)	0.700***
Minimum value per 6 areas, median (IQR), kPa	3.85 (3.13–5.21)	3.21 (2.60–5.06)	0.203***
Minimum value per 9 areas, median (IQR), kPa	3.75 (2.93–4.96)	3.05 (2.16–3.95)	0.040***
Maximum value per 3 areas, median (IQR), kPa	6.37 (4.81–9.86)	8.34 (4.18–20.70)	0.144***
Maximum value per 6 areas, median (IQR), kPa	7.55 (5.83–10.65)	12.05 (7.62–20.70)	0.009***
Maximum value per 9 areas, median (IQR), kPa	8.69 (6.57–11.48)	13.15 (8.06–21.40)	0.008***
Range per 3 areas, median (IQR), kPa	1.80 (1.08–3.44)	3.40 (0.99–7.96)	0.043***
Range per 6 areas, median (IQR), kPa	3.55 (2.41–5.44)	6.83 (4.14–15.89)	0.003***
Range per 9 areas, median (IQR), kPa	4.72 (3.00–6.46)	8.64 (5.01–17.57)	0.001***

*NPP* normal pancreatic parenchyma, *PC* pancreatic cancer, *IQR* interquartile range.

*Mann–Whitney U test.

## Discussion

In this study, we investigated the usefulness of SWE as an objective and quantitative evaluation method for PC and found that there was no difference in the elastic modulus measured in PC and NPP. However, the variability of elastic modulus within each patient was significantly greater in PC than in NPP.

Elastography of the pancreas has been studied in various ways. Itoh et al.^14^ found that elastography using EUS (EUS-EG) is useful in the diagnosis of pancreatic fibrosis. Kuwahara et al.^15^ also found that EUS-EG is useful for the objective evaluation of chronic pancreatitis. Some reports on the usefulness of EUS-EG for the qualitative evaluation of pancreatic tumours are available^16–19^, but most studies on elastography of the pancreas are conducted using EUS, and only a few reports show that elastography using transabdominal US is useful for the evaluation of pancreatic tumours^8^. The reason for this is that the pancreas is located deep inside the body, and in some cases, it is difficult to obtain a stable image with transabdominal ultrasound. Compared to strain elastography, which uses the external force generated in the body, SWE has a fixed shear wave for each instrument and is more repeatable and quantifiable than the former. SWE has already been reported to be useful in the evaluation of liver fibrosis and steatosis, and the optimal number of measurements and other procedures are being established^6^. However, it is not known in detail whether reproducible measurement values can be obtained for the pancreas or what the optimal measurement conditions are. Furthermore, although SWE is a quantitative evaluation, the measurement values obtained may differ depending on the instrument^10^, and we believe it is necessary to evaluate the measurement conditions for each instrument.

In this study, we first examined the optimal number of measurements of the ElastQ mode of the EPIQ 7G used in this study. It was shown that appropriate SWE values were obtained by measuring SWE in three images, meaning that nine measurements were performed. Conventional SWE is point shear wave elastography (pSWE), which is a technique to obtain a single measurement value at any point by referring to the B-mode. However, 2D-SWE, which has been installed in the high-end models of each instrument in recent years, is capable of measuring a wide range of areas at the same time, and it is possible to make appropriate measurements intuitively and visually by displaying a colour map. Kuwahara et al.^20^ reported that the number of measurements required for pSWE reproducibility was five, while Hashizume et al.^21^ reported that the number of measurements required to obtain reliable data using the propagation display of 2D-SWE was three. In this study, the optimal number of measurements obtained was three by using the stiffness map and IQR/median as a reference to determine the measurement locations. 2D-SWE improves the reproducibility and is considered to be a technique that can be applied to the pancreas.

Very few reports have evaluated the stiffness of PC using SWE, but the available reports indicate that PC is stiffer than normal pancreatic tissue. Zaro et al.^22^ found that the mean SWE of PC was significantly higher than that of healthy pancreas tissue in a pilot study of 33 patients, including 18 controls with healthy pancreases and 14 PC (adenocarcinoma) patients. D'Onofrio et al.^23^ found that the median SWE was significantly higher in 62 PC (adenocarcinoma) patients than in 40 individuals with healthy pancreases. Additionally, by strain elastography, PC is considered a stiffer tissue than normal pancreatic tissue and other surrounding tissue^8^. In the present study, the median elastic modulus was not significantly higher in PC cases, but the variability of the elastic modulus inside the same lesion was significantly greater in PC cases. PC tumours are known to be characterized by molecular pathological heterogeneity^12^, and such histological heterogeneity may have influenced the measurement results. In the present study, we focused on the minimum value, maximum value and range of elastic modulus to evaluate the variability. The minimum elastic modulus value in PC was significantly smaller than NPP with nine measurements (*P* = 0.040, respectively), and the maximum elastic modulus value in PC was also significantly higher than NPP with six and nine measurements (*P* = 0.009, *P* = 0.008, respectively). These results indicate that PC consists of stiffer tissues than NPP but also contains soft tissues with elastic modulus similar to that of NPP. The range was also significantly greater in PC with three, six, and nine measurements (*P* = 0.043, *P* = 0.003, *P* = 0.001, respectively). The specificity of the ROC curve using the range was 84.5% with a cut-off value of 7.88 kPa, the positive predictive value was 50%, and the negative predictive value was 88.8%. Considering the high negative predictive value described above, when a lesion demonstrates high elastic modulus but low variability, the possibility of a non-cancerous lesion should be considered. Other pancreatic tumours, such as pancreatic neuroendocrine tumours and solid pseudopapillary neoplasms, are considered to be more homogeneous tumours than PC^24–27^ and have less variation from case to case. Although other pancreatic tumours were not investigated in this study, quantitatively evaluating the histological characteristics of tumours by measuring SWE in other solid pancreatic tumours may be possible. It may also be useful in differentiating PC and inflammatory changes such as MFP. In this study, although there were no differences in the median elastic modulus between the two groups, the variability of MFP was significantly smaller than that of PC. Because of the small number of MFP cases, it may be difficult to conclude that SWE is useful in differentiating PC and MFP from our results, but we believe that we have shown the potential usefulness.

There are some limitations in this study. First, there were no pathological diagnoses in the control group, so there may have been a mixture of pancreatic diseases that were not detectable from the history and imaging findings. Second, this study did not include other pancreatic tumours. Third, this was a single-center, retrospective observational study with a small number of patients, particularly the patients with PC and MFP, and the findings need to be investigated in a larger cohort of prospective, multicenter studies in the future including other pancreatic tumours.

## Conclusions

2D-SWE showed that elastic modulus measured was not different between PC and NPP, but the variability of elastic modulus was significantly greater in PC than in NPP. Evaluating the obtained elastic modulus itself is not useful in differentiating PC from NPP. However, evaluating the range of elasticities that can be obtained relatively easily will provide additional information in SWE, which may be useful in the diagnosis of PC.

## Data Availability

The datasets generated and analysed during the current study are available from the corresponding author on reasonable request.

## Abbreviations

SWE: Shear wave elastography
SWS: Shear wave speed
PC: Pancreatic cancer
NPP: Normal pancreatic parenchyma
BMI: Body mass index
IQR: Interquartile range
EUS: Endoscopic ultrasound
EUS-FNA: Endoscopic ultrasound-guided fine-needle aspiration
2D-SWE: Two-dimensional shear wave elastography
ICC: Intraclass correlation coefficient
EUS-EG: Elastography using endoscopic ultrasound
pSWE: Point shear wave elastography
