# Author Correction: Variability measurements provide additional value to shear wave elastography in the diagnosis of pancreatic cancer

**DOI:** 10.1038/s41598-022-21219-y

**Published:** 2022-09-28

**Authors:** Masakatsu Yoshikawa, Takuya Ishikawa, Eizaburo Ohno, Tadashi Iida, Kazuhiro Furukawa, Masanao Nakamura, Takashi Honda, Masatoshi Ishigami, Fumie Kinoshita, Hiroki Kawashima, Mitsuhiro Fujishiro

**Affiliations:** 1 Department of Gastroenterology and Hepatology, Nagoya University Graduate School of Medicine, 65 Tsuruma‑cho, Showa‑ku, Nagoya, Aichi 466‑8550 Japan; 2 Data Coordinating Center, Department of Advanced Medicine, Nagoya University Hospital, 65 Tsuruma‑cho, Showa‑ku, Nagoya, Aichi 466‑8550 Japan; 3 Department of Endoscopy, Nagoya University Hospital, 65 Tsuruma‑cho, Showa‑ku, Nagoya, Aichi 466‑8550 Japan

Correction to: *Scientific Reports* 10.1038/s41598-021-86979-5, published online 01 April 2021

The original version of this Article contained an error. In the Materials and methods section, under the subheading ‘Study design’, the approval number of the Ethics Committee was incorrect.

As a result,

“The study was conducted with the approval of the Ethics Committee of Nagoya University Hospital and enrolled in the jRCT (CRB4180004).”

now reads:

“The study was conducted with the approval of the Ethics Committee of Nagoya University Hospital (approval number: 2014-0399).”

The original Article has been corrected.

